# Integrating Psychological Support and Topical Therapy for the Effective Management of Stress-Induced Alopecia Areata: A Case Report

**DOI:** 10.7759/cureus.77317

**Published:** 2025-01-12

**Authors:** Amna A Alotiby

**Affiliations:** 1 Department of Haematology and Immunology, College of Medicine, Umm Al-Qura University, Makkah, SAU

**Keywords:** alopecia areata, autoimmune, combined therapy, minoxidil, stress-induced alopecia areata, stress-reduced therapy

## Abstract

Alopecia areata is an autoimmune disorder characterized by hair loss in defined patches. Genetic factors are considered primary contributors to the onset and exacerbation of certain conditions. However, environmental triggers, particularly psychological stress, also play a significant role. This case report presents a 28-year-old woman with no prior history of autoimmune or immune-related conditions who developed severe alopecia areata following prolonged emotional stress. Despite the absence of systemic or nutritional deficiencies, she experienced significant hair loss, which improved with 5% Minoxidil and psychological stress management. However, a relapse occurred after another period of acute stress, highlighting the relationship between psychological stress and disease progression. This case underscores the need for a comprehensive treatment approach, combining pharmacological interventions like Minoxidil with stress reduction strategies to manage stress-induced autoimmune disorders and prevent relapses effectively. Such a combined approach is crucial in treating and managing alopecia areata, especially in patients with psychological stress triggers.

## Introduction

Alopecia areata (AA) is an autoimmune disorder that predominantly causes non-scarring hair loss, typically presenting as well-circumscribed, round patches. Although the precise etiology of AA remains incompletely understood, a complex interplay of genetic, immunological, and environmental factors is believed to contribute to its onset. Autoimmune mechanisms, where the individual's immune system erroneously targets its hair follicles, are central to its pathophysiology [1]. Stress, both acute and chronic, has been increasingly recognized as a trigger for alopecia areata, especially in patients who have a genetic predisposition to autoimmune diseases [2]. The exact mechanisms through which stress induces AA are still being explored, but it is widely accepted that stress can dysregulate immune function, disrupt hair follicle growth, and activate inflammatory pathways [3].

The mechanism linking stress to AA is thought to involve the activation of the hypothalamic-pituitary-adrenal (HPA) axis, which releases cortisol and other stress-related hormones. These hormones can affect immune cell function, influencing cytokine production and activating inflammatory pathways that lead to the attack on hair follicles [4]. Psychological stress has been shown to increase the release of pro-inflammatory cytokines, such as interferon-gamma, which plays a crucial role in the autoimmune destruction of hair follicles [3].

The pathogenesis of AA involves a T lymphocyte-mediated autoimmune response, where infiltrating CD8+ T-cells promote an inflammatory cascade that impairs normal hair follicle cycling and disrupts hair growth [5]. Recent studies have elucidated that stress may exacerbate or trigger AA through its modulatory effects on immune function, thereby promoting the activation of T-cells and the initiation of inflammatory processes [3,4].

In this context, CD8+ T-cells undergo a phenotypic shift from an immunosuppressive to an inflammatory state, secreting interferon-alpha (IFN-α), which induces premature follicular entry into the catagen phase [3]. Additionally, hair follicles express MHC class I molecules, which are typically downregulated to maintain their immune-protected environment. However, under stress, the release of substance P upregulates MHC class I expression, thereby disrupting this protective state and facilitating immune-mediated damage to the hair follicles. Remarkably, the hair follicles' immune protection appears to be restored following the cessation of psychological stress [6]. Although apoptosis is considered a secondary pathway in the pathogenesis of AA, it is thought to complement the autoimmune mechanism. Substance P, which is released during psychological stress, downregulates the expression of tropomyosin receptor kinase A (TrkA) while upregulating the p75 neurotrophic receptor. These molecular changes lead to the activation of apoptotic pathways within the keratinocytes of hair follicles, further contributing to hair follicle damage and hair loss [7,8].

A variety of therapeutic modalities are currently employed for the management of AA, including corticosteroids, topical immunotherapy, and Minoxidil [1]. However, the influence of stress management on preventing relapse or mitigating the severity of the condition remains insufficiently explored in the literature. Psychological stress is a significant modifiable risk factor in the onset, exacerbation, and progression of immune-related disorders such as AA [3,4]. Therefore, effective stress management strategies may play a pivotal role in enhancing therapeutic outcomes and reducing the recurrence of the disease.

The present case report is the first to explore the synergistic effects of combining pharmacological treatment with 5% Minoxidil and targeted stress reduction interventions in the management of stress-induced AA. The report underscores the potential benefits of integrating stress management strategies into the standard therapeutic regimen for AA. It posits that such an integrative approach may augment the clinical response, foster hair regrowth, and contribute to prolonged remission in affected individuals. This integrated model may serve as an essential framework for the management of AA, advocating for the inclusion of psychological interventions in routine clinical care, particularly for patients with stress-induced or stress-exacerbated forms of the disorder.

## Case presentation

A 28-year-old female with no significant past medical history or family history of autoimmune conditions presented to the dermatology clinic with complaints of hair loss. The patient described experiencing a prolonged period of severe emotional stress lasting more than six months, attributed to personal, familial, and professional challenges. She had never experienced stress of such magnitude in the past. Approximately one month after the stressor was alleviated, the patient noticed two distinct patches of hair loss on the left side of her scalp, both oval in shape and measuring approximately 3 cm in diameter.

Physical examination and diagnosis

On physical examination, the patient presented with two well-defined, non-scarring patches of alopecia on the left temporal scalp. There were no signs of inflammation, erythema, or tenderness in the affected area. No evidence of other systemic involvement, such as nail abnormalities or signs of systemic autoimmune disease, was noted. The remainder of the scalp and body appeared normal. Laboratory investigations, including thyroid function tests, complete blood count (CBC), iron panel, and serum ferritin, all returned within normal limits (Table 1). Serum ferritin levels were 20 ng/mL, which, while low, did not indicate iron deficiency anemia, as other parameters, such as serum iron, total iron-binding capacity (TIBC), mean corpuscular volume (MCV), and mean corpuscular hemoglobin (MCH), were all within the normal range. Blood analysis revealed no autoimmune markers, effectively ruling out other potential causes of hair loss such as systemic lupus erythematosus or thyroid dysfunction. The temporal association between the onset of hair loss and the prolonged period of stress, combined with the absence of other underlying causes, led to a clinical diagnosis of alopecia areata. In light of the patient's lack of a family history of autoimmune disease, this presentation was consistent with stress-induced AA, which is increasingly recognized in both clinical practice and research as a key trigger for the condition.

**Table 1:** Summary of blood analysis

Test	Result	Normal Range
Hemoglobin	13.5 g/dL	12.0 - 15.5 g/dL
Red Blood Cell (RBC) Count	5 x 10^6/µL	4.2 - 5.4 x 10^6/µL
Mean Corpuscular Volume (MCV)	90 fl	84-96 fl
Mean Corpuscular Hemoglobin (MCH)	30 pg	28-34 pg
TSH (Thyroid Stimulating Hormone)	1.7 µIU/mL	0.5 - 5.0 µIU/mL
Free T3	3.3 pg/mL	2.3 - 4.2 pg/mL
Free T4	0.82 ng/dL	0.8 - 1.8 ng/dL
Serum Ferritin	20 ng/mL	30 - 400 ng/mL
Serum Iron	50 ug/dl	33-193 ug/dl
Total Iron Binding Capacity (TIBC)	250 ug/dl	228-428 ug/dl

Initial management and treatment plan

Following the clinical diagnosis of AA and its association with psychological stress, the patient was prescribed Minoxidil 5% topical solution for use twice daily on the affected areas. Minoxidil, a Food and Drug Administration (FDA)-approved therapy for AA, promotes hair regrowth by improving blood circulation to the hair follicles, stimulating follicular activity, and prolonging the anagen (growth) phase of the hair cycle. The patient was counseled on the expected timeline for regrowth, emphasizing that the response to treatment could take several months, as hair follicles require time to regenerate.

In addition to pharmacological treatment, the patient was encouraged to implement stress management strategies to address the psychological factors contributing to the onset and progression of her condition. These interventions included self-directed mindfulness meditation, which the patient engaged in regularly to manage the chronic psychological stress associated with AA. The patient was also advised to strengthen her emotional support system by seeking the support of her family, which was identified as a crucial factor in maintaining emotional well-being during treatment. Additionally, the patient was encouraged to participate in volunteer community service, providing her with a sense of purpose and connection that further enhanced her overall mental health. To further support hair regrowth and improve the health of newly formed hair, the patient was prescribed iron supplementation, which could help support follicular health and improve the efficacy of Minoxidil.

Treatment response and follow-up

At the three-month follow-up visit, the patient reported significant improvement in hair regrowth and her emotional state. The patches of alopecia had visibly filled in with fine, soft hair, and there was a noticeable improvement in hair density across the affected areas. The patient also noted a marked reduction in her stress levels, facilitated by her consistent adherence to stress management strategies and support from her family. Psychological improvements were reflected in her reported emotional state and her reduced perception of stressors, suggesting that the combination of stress management and Minoxidil treatment contributed synergistically to the positive outcome.

However, one year after completing the initial course of treatment, the patient stopped the stress management program and experienced another episode of acute emotional stress related to a new personal crisis. During the onset of stress, she noticed new patches of non-scarring alopecia on the frontal region of her scalp. She returned to her dermatologist for re-evaluation, and upon confirming the recurrence of alopecia areata, the same treatment regimen of Minoxidil 5% was re-initiated. The patient was once again advised to follow her stress management plan. After three months, she again experienced significant regrowth in the affected areas, and her stress levels improved with continued psychological support.

## Discussion

Pathophysiology of stress-induced alopecia areata

In our patient, severe, long-term stress lasting more than six months can be considered the primary causative factor behind the hair loss. The patient's medical history and relevant laboratory investigations were unremarkable, ruling out other physiological causes of the condition. Additionally, there was no family history of conditions associated with hair loss, thus ruling out a genetic predisposition to alopecia areata. The onset of hair loss symptoms immediately after the stressful event and the reappearance of symptoms upon exposure to the same stressor strongly suggest a causal relationship based on temporal association and rechallenge. Finally, stress relief techniques, in addition to medication, proved effective, resulting in a favorable treatment response and faster healing.

The pathophysiology of stress-induced AA is multifactorial, involving both autoimmune and neurogenic pathways. Stress activates the HPA axis, leading to the release of cortisol, which influences immune function and promotes inflammatory cytokines, such as interferon-gamma, contributing to hair follicle damage [9,10]. Additionally, stress-related neuropeptides, like Substance P (SP) and corticotropin-releasing hormone (CRH), are involved in the release of inflammatory mediators, exacerbating the autoimmune attack on hair follicles [11,12].

Our findings align with similar studies, such as Ahn et al. (2023), which explored the role of stress-related neuropeptides (SP and CRH) in the pathogenesis of AA, highlighting the temporal association between stress and the onset of the disease [3]. Furthermore, Propp et al. (2024) found that repeated acute stress influences immune responses and the HPA axis, correlating with our observations of immune system involvement in stress-induced AA [13]. Thus, the present case contributes to the growing evidence base indicating that stress, through both immune-mediated and neurogenic mechanisms, plays a significant role in the pathogenesis and exacerbation of AA.

The correlation between blood parameters and AA is essential for understanding its pathophysiology and treatment outcomes. In this case, the patient’s serum ferritin level of 20 ng/mL, though below the normal range, does not meet the criteria for iron deficiency anemia, as other markers, such as serum iron and TIBC, remain normal. However, low ferritin levels can still contribute to increased hair loss, as iron is vital for hair follicle function. Previous research indicates that iron deficiency, even without anemia, may exacerbate AA, suggesting that iron repletion could improve treatment outcomes [14,15].

Thyroid function tests (normal TSH, Free T3, and Free T4) rule out thyroid dysfunction as a cause of hair loss, consistent with studies linking thyroid abnormalities to worsened hair thinning in AA [16]. While hemoglobin and RBC counts are normal, anemia could still impede blood flow to the scalp, exacerbating hair loss [15].

Overall, the findings indicate that the patient's AA is primarily stress-induced, with no significant contribution from hematologic or thyroid disorders. Low ferritin levels, though not indicating iron deficiency anemia, may still contribute to the severity of the condition, suggesting that iron repletion may be beneficial in the management of AA.

Treatment modalities and the role of Minoxidil

The treatment of alopecia areata has evolved significantly, with topical corticosteroids, immunotherapy, and Minoxidil emerging as the primary pharmacologic therapies. Minoxidil, a well-established treatment, promotes hair regrowth by stimulating hair follicles and enhancing blood flow to the scalp. Although the exact mechanism of action remains unclear, it is believed to increase follicular blood supply, prolong the anagen phase of the hair cycle, and enlarge miniaturized hair follicles [17]. In this case, the patient's positive response to Minoxidil suggests that pharmacologic treatment can be effective in addressing hair loss, particularly when linked to stress-induced alopecia. Studies have consistently shown that Minoxidil, especially at a 5% concentration, yields a substantial therapeutic response in promoting hair regrowth [17,18]. This aligns with the patient's positive outcomes, reinforcing its role as an effective treatment for AA. In comparison, topical corticosteroids are often used as a first-line treatment for mild to moderate AA, with intralesional corticosteroids being particularly beneficial in cases of extensive scalp involvement [18]. Although both treatments are effective, Minoxidil may be preferred by patients seeking non-steroidal options, as was the case here.

However, Minoxidil alone may not fully address the underlying causes of stress-induced alopecia. The recurrence of alopecia following subsequent emotional stress in this patient underscores the importance of an integrated treatment approach. While Minoxidil effectively promotes hair regrowth, it may not prevent relapses triggered by psychological factors. Therefore, incorporating stress management strategies is essential for long-term management. Mindfulness, emotional support, and coping mechanisms are integral components in addressing the psychological contributors to AA [3,19].

Despite its therapeutic benefits, Minoxidil requires consistent application to maintain hair regrowth, and discontinuation may lead to a recurrence of hair loss in some AA patients. Furthermore, potential side effects, such as scalp irritation, dryness, and unwanted hair growth in surrounding areas, are important considerations [18]. These limitations emphasize the need for patient education, adherence to treatment protocols, and an integrated approach that addresses both the physical and psychological aspects of alopecia areata.

In conclusion, Minoxidil remains a cornerstone in the pharmacologic management of AA, particularly in cases linked to stress. However, for optimal results, it should be combined with stress management strategies to address the root psychological triggers, ensuring a comprehensive and effective treatment plan.

Psychosocial interventions in the management of AA

Psychological stress is a well-documented trigger for alopecia areata, and managing stress is crucial in preventing relapses. Cognitive-behavioral therapy (CBT), mindfulness-based stress reduction (MBSR), and other psychological interventions have been shown to improve the outcomes of individuals with AA, particularly in those with stress-related or stress-exacerbated forms of the disease [3,19]. In this case, the patient was encouraged to practice mindfulness meditation, which has been shown to reduce stress and improve overall well-being in individuals with chronic conditions, including AA. These findings align with studies demonstrating that mindfulness meditation effectively reduces stress and improves the quality of life for individuals with dermatologic conditions [3,19].

This case report is the first to highlight the importance of addressing both the physical and emotional components of stress-induced AA management. The patient's significant improvement with the combination of Minoxidil and stress management techniques underscores the need for a holistic approach to treatment.

## Conclusions

To the best of the author’s knowledge, this case report is the first to highlight the significant role that stress plays in the onset and exacerbation of alopecia areata (AA) and underscores the potential benefits of a combined treatment approach. The patient’s experience demonstrates that while pharmacologic therapies, such as Minoxidil, can effectively promote hair regrowth, addressing the psychological factors contributing to AA through stress management strategies is essential for long-term success. The temporal association between stress and hair loss, as well as the recurrence of alopecia following stress exposure, supports the hypothesis that stress plays a pivotal role in both the initiation and relapse of the condition. The patient’s positive response to an integrated treatment regimen, comprising Minoxidil and targeted stress reduction techniques, further emphasizes the importance of incorporating psychological interventions as a standard part of care for patients with stress-induced or stress-exacerbated AA. Future research exploring the impact of stress management on clinical outcomes in AA patients may provide valuable insights into optimizing therapeutic strategies, ultimately fostering better long-term results and enhancing the quality of life for those affected by this challenging condition.
